# Light Chain Diversity among the Botulinum Neurotoxins

**DOI:** 10.3390/toxins10070268

**Published:** 2018-07-02

**Authors:** Alexander P. Gardner, Joseph T. Barbieri

**Affiliations:** Microbiology and Immunology, Medical College of Wisconsin, 8701 Watertown Plank Rd., Microbiology and Immunology, BSB-255, Milwaukee, WI 53226, USA; agardner@mcw.edu

**Keywords:** botulinum neurotoxins, SNARE proteins, zinc metalloproteases, neurons, toxins, synaptic vesicles

## Abstract

Botulinum neurotoxins (BoNT) are produced by several species of clostridium. There are seven immunologically unique BoNT serotypes (A–G). The Centers for Disease Control classifies BoNTs as ‘Category A’ select agents and are the most lethal protein toxins for humans. Recently, BoNT-like proteins have also been identified in several non-clostridia. BoNTs are di-chain proteins comprised of an *N*-terminal zinc metalloprotease Light Chain (LC) and a C-terminal Heavy Chain (HC) which includes the translocation and receptor binding domains. The two chains are held together by a disulfide bond. The LC cleaves Soluble *N*-ethylmaleimide-sensitive factor attachment protein receptors (SNAREs). The cleavage of SNAREs inhibits the fusion of synaptic vesicles to the cell membrane and the subsequent release of acetylcholine, which results in flaccid paralysis. The LC controls the catalytic properties and the duration of BoNT action. This review discusses the mechanism for LC catalysis, LC translocation, and the basis for the duration of LC action. Understanding these properties of the LC may expand the applications of BoNT as human therapies.

## 1. Background on Botulinum Neurotoxin

Botulinum neurotoxins (BoNT), the causative agents of botulism, are produced by several species of *Clostridium*, including *botulinum*, *baratii*, and *butyricum*. Clostridia are Gram-positive, spore-forming, anaerobic bacteria [1,2,3]. There are seven immunologically different serotypes of BoNT (A–G), categorized based upon immunity cross protection [4]. Using bioinformatic tools, several new BoNT derivatives have been identified, including BoNT/FA, BoNT/en (eBoNT/J) [5,6], BoNT/Wo [7], and BoNT/X [8], which represent potentially new BoNT serotypes. Other references that discuss and/or analyze sequence variation in the BoNT light chain include Ref. [9,10].

Select agents are “agents and toxins that have been determined to have the potential to pose a severe threat to public health and safety, to animal and plant health, or to animal or plant product” [11]. The CDC has catalogued select agents and toxins into three Categories A, B, and C, where Category A agents and toxins pose the greatest risk to national security [12]. There are six ‘Category A’ agents: Anthrax, Botulism, Plague, Smallpox, Tularemia, and viral hemorrhagic fevers such as Ebola, Marbury, and Dengue [11,13,14]. In contrast, tetanus toxin and diphtheria toxin are not cataloged as a risk in the United States and other developed countries due to routine vaccination with tetanus and diphtheria toxoids [15]. 

BoNTs are physically organized as a *N*-terminal catalytic domain termed Light Chain (LC) and a *C*-terminal translocation/receptor binding domain termed Heavy Chain (HC). This review will describe our current understanding of the biological and biochemical action of the LC.

## 2. BoNT Structure and Function

BoNT are synthesized as single chain proteins which are typically isolated as di-chain forms [16], except BoNT/E which is isolated as a single chain toxin and cleaved by host proteases into the di-chain form [16,17,18]. Di-chain BoNT/E is ~100-fold more toxic than in the single chain form [18], showing that di-chain cleavage is a step in the BoNT activation process. BoNTs are tri-domain proteins that are 1300 amino acids in length. In the di-chain form, BoNTs are classified as AB toxins. The A subunit (50 kDa) is the catalytically active LC. The B subunit (100 kDa) is the HC, which contains the translocation domain (HCN) and the receptor binding domain (HCC). The A and B subunit are covalently linked by a disulfide bond [19,20,21].

The HCC is 50 kDa and binds polysialoganglioside (gangliosides containing sialic acids) enriched on the presynaptic membrane exposed to the extracellular environment. Upon membrane depolarization, the HCC will bind to the luminal domain of the Synaptic Vesicle protein 2 (SV2), or synaptotagmin and is endocytosed into a synaptic vesicle [22,23]. As the synaptic vesicle matures, a Na^+^-K^+^ ATPase reloads synaptic vesicles with neurotransmitter molecules into the acidified vesicle lumen. As the vesicle acidifies, at ~pH 4.5 a conformational change occurs in the HCN and embeds into the membrane of the synaptic vesicle forming a pore of ~15 Å in diameter for disulfide bond reduction and LC translocation into the cytosol [24,25]. The translocation mechanism is not fully understood, but if similar to diphtheria toxin, translocation initiates at the C terminus of the LC [26]. The HCN then aids in the refolding of the cytosolic LC [27]. Once refolded in the cytosol, LC then anterograde traffics to the membrane to cleave the appropriate substrate.

**Figure 1:**
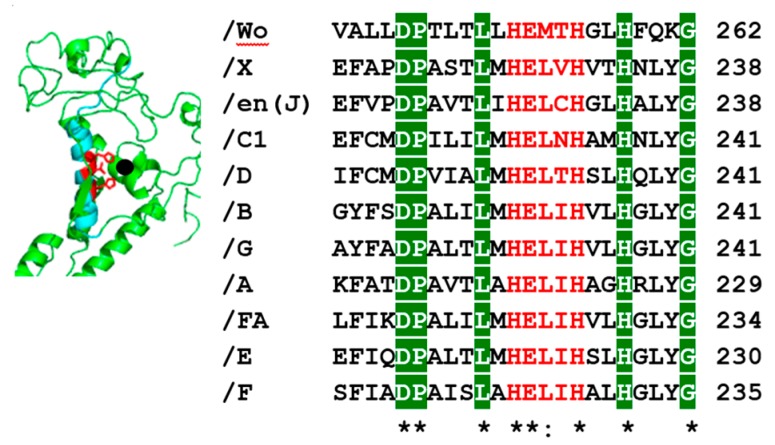
Clostridial BoNT and non-clostridial BoNT derivatives. (Left) Structure and alignment (Right) of the conserved zinc binding motif H-E-X-X-H in red lettering. Residues in highlighted in green are conserved. Accession numbers BoNT A-G from UniProt BoNT/A: P10845, BoNT/B: P10844, BoNT/C1: P18640, BoNT/D: P19321, BoNT/E: Q00496, BoNT/F: P30996 and BoNT/G: Q60393. BoNT/FA: GenBank (KGO15617.1), BoNT/X: UniParc (UPI0005822796), BoNT/Wo: UniProtK (A0A069CUU9 (A0A069CUU9_WEIOS)) and BoNT/en: GenBank (OTO22244.1).

**Figure 2:**
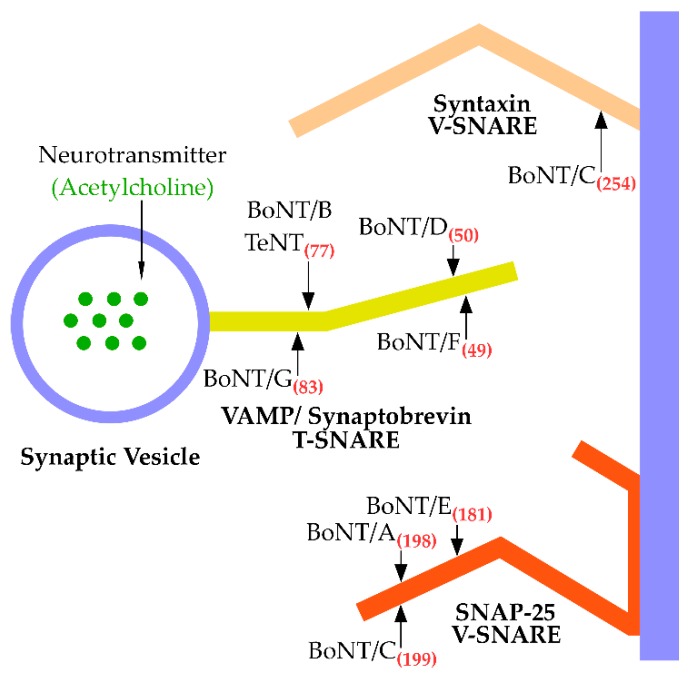
BoNT serotype (A–G) SNARE cleavage. BoNT cleavage prevents interaction of the T-SNARE and V-SNARE, preventing acetylcholine release. BoNT/A and /E cleave SNAP-25, BoNT/C cleaves SNAP-25 and Syntaxin which are located on the cytosolic face of the cell membrane. BoNT/B, /D, /F, /G, and tetanus toxin (TeNT) cleave VAMP/ Synaptobrevin which is located on the cytosolic face of synaptic vesicles. In red are the amino acid # of the P1′ scissile bond cleavage by the indicated BoNT serotype.

A subset of zinc metalloproteases, including BoNT, contain a consensus sequence His-Glu-X-X-His (Figure 1) [28,29]. BoNT belongs to the thermolysin family of zinc metalloproteases [30]. BoNTs cleave Soluble *N*-ethylmaleimide-sensitive factor Attachment protein REceptors (SNARE) where each BoNT serotype cleaves a unique site within a SNARE protein or multiple SNARE proteins [31]. Light Chain A (LC/A) and LC/E cleave (Figure 2) synaptosomal-associated protein (SNAP-25) [32,33,34]. LC/C (Figure 2) also cleaves SNAP-25 and two isoforms of Syntaxin (Syntaxin-1A and Syntaxin-1B), a T-SNARE [33]. Light Chain of Tetanus toxin (LC/T), LC/B, LC/D, LC/F, LC/FA and LC/G (Figure 2) cleave vesicle-associated membrane protein (VAMP)/Synaptobrevin, a V-SNARE [32,35,36,37,38]. BoNT/FA, previously named as a new serotype, BoNT/H, is a chimera between BoNT/F and BoNT/A [39]. LC/FA shares 81% homology with LC/F5, while the HCC/FA has 93% identity to BoNT/A1 [40]. Among the non-clostridia BoNT-like proteins, BoNT/X cleaves VAMP1-3 at a unique site, and cleaves VAMP 4 and 5, as well as Ykt6 (a VAMP family protein). LC/X is also ~10 fold more efficient at cleavage of VAMP1 with a turnover rate of 271 min^−1^ compared to LC/B, which has a turnover rate of 47 min^−1^ [41]. Another non-clostridia LC/en cleaved VAMP 1-3, Syntaxin (Syx 1B and Syx 4), as well as SNAP-23 and SNAP-25, albeit with a low efficiency for SNAP-25 and Syx 1B and Syx 4 was not cleaved. At high concentrations, LC/en cleaved Syx 4 [5]. LC/Wo cleaves VAMP-2 [7].

In neurons, SNARE proteins on synaptic vesicles and on the cytosolic face of the plasma membrane constitute the mechanism machinery that initially engages synaptic vesicles with the plasma membrane and then coil to facilitate the fusion of the synaptic vesicle into the plasma membrane to allow the release of neurotransmitters at the neuromuscular junction [42]. LCs recognize SNARE protein lengths of 16 to >50 amino acids, while other zinc proteases cleave peptides as short as two amino acids [30]. SNARE protein cleavage inhibits neurotransmitter, acetylcholine, release, which inhibits muscle contraction at the neuromuscular junction [16]. This leads to the flaccid paralysis characteristic of botulism [43]. This extended region of sequence recognition helps define the neuronal specificity of BoNTs as neurotrophic toxins, by reducing the possibility of off-target cleavage of non-neuronal isotype SNARE proteins. This adds to the utility of BoNTs as therapeutic agents. 

## 3. Cleavage of BoNT Substrates

Several groups have studied the sites recognized by each of the seven BoNT serotypes, as well as the non-clostridial BoNT derivatives for cleavage of various SNARE proteins. Binz et al. used several techniques, such as radiolabeled SNAP-25, HPLC, and microsequencing, to identify the cleavage site of LC/A and LC/E. Incubation of radiolabeled SNAP-25 with the LC/A yielded a nanopeptide corresponding to the C terminus SNAP-25 between residues E^197^ and R^198^. Using the same techniques, LC/E cleaved between residues R^180^ and I^181^ [44]. More recent experiments show in a competition experiment between LC/A and LC/E, LC/E outcompeted LC/A for cleavage of SNAP-25, due to LC/E cleavage site being upstream of LC/A [45].

BoNT/C is the only BoNT serotype to cleave Syntaxin-1A between K^253^ and A^254^ and Syntaxin-1B between K^252^ and A^253^ [46]. Another group showed LC/C also cleaved SNAP-25 adjacent to the bond that BoNT/A cleaves, between residues R^198^ and A^199^ [47]. LC/B, LC/D, LC/F, and LC/G cleave the vesicle-bound SNARE protein VAMP/Synaptobrevin. Schiavo et al. showed BoNT/B inhibited acetylcholine release by proteolytic cleavage of synaptobrevin between E^76^ and F^77^ and showed tetanus toxin, a related clostridial neurotoxin, cleaved synaptobrevin between the same residues, but in inhibitory neurons which led to spastic paralysis [35]. Yamasaki et al. used radiolabeled *Aplysia* synaptobrevin to show LC/D and LC/F cleaved synaptobrevin between K^49^ and I^50^ and E^48^ and K^49^, respectively [36]. BoNT/FA cleaved the same substrate as BoNT/F5 between residues L^54^ and E^55^ on synaptobrevin. Other BoNT/F subtypes cleaved synaptobrevin between E^58^ and K^59^ [37,48,49,50,51]. BoNT/G cleaved synaptobrevin-1 between A^82^ and A^83^ and synaptobrevin-2 between A^81^ and A^82^ [38]. 

BoNT/X cleaves VAMP 1-5 and Ykt6 between R^66^ and A^67^. Cleavage of VAMP-4, VAMP-5 and Ykt6 is unique to BoNT/X [41]. BoNT/en cleaved VAMP-2 between A^67^ and D^68^, Syx 1B between M^182^ and D^183^, and Syx 4 between K^191^ and D^192^. Zhang et al. were not able to determine the cleavage site of SNAP-23/25 due to multiple smeared bands of substrate arising upon incubation with high LC/en concentrations [5]. BoNT/Wo cleaved close to the C terminus of VAMP-2, within the juxtamembrane domain between residues W^89^ and W^90^ [7]. Overall, the specificity for substrate is greater for the BoNTs, relative to the non-clostridial BoNT-like proteins, suggesting that the evolution of the neurotrophic nature of the BoNTs included an enhanced specificity for substrate recognition. Future studies may reveal the molecular basis for the differential substrate specificity that may provide insight into the steps required for the modulation of LCs to customize substrate cleavage. 

## 4. Exosite-Pocket Model for LC-Mediated SNARE Cleavage 

**Figure 3:**
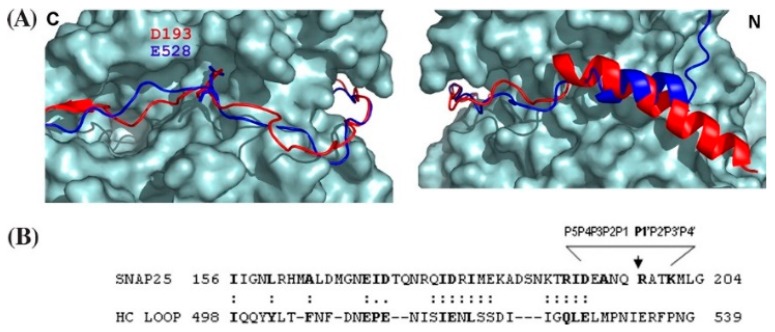
Structural alignment of BoNT/A heavy chain loop with SNAP-25. (**A**) Structural alignment of the HC loop of BoNT/A and SNAP-25 with LC/A. Left panel shows the alignment from the C terminus of the HC loop of BoNT/A and SNAP-25 on the face of LC/A that continues in the right panel along the back of LC/A toward the N termini. The blue ribbon represents the HC loop; the red ribbon represents SNAP-25. Asp193 of SNAP-25 and Glu528 of the HC loop interactions with LC/A S5 pocket ends the similarity between SNAP-25 and the HC loop of BoNT/A; (**B**) Sequence and spatial similarity of HC loop of BoNT/A and SNAP-25 interacting with LC/A. The spatial overlap of the HC loop of BoNT/A and residues of SNAP-25 that interact with LC/A is indicated by (:) or partial overlap (.), which was determined by manual alignment of the two structures. Note the structural overlap between the HC loop of BoNT/A and SNAP-25 ends at Asp193. Reproduced from [53]. Copyright 2007, American Society for Biochemistry and Molecular Biology, Rockville, MD, USA.

Unexpectedly, SNAP-25 binds to the grove within LC/A, which is also the binding region of the HCN belt to the LC. The HCN belt comprises residues 458–547 of the HC (Figure 3) [52,53,54]. The HCN belt region and SNAP-25 align along a stretch of 38-amino acids which aligned within the LC/A crystal structure (Figure 3). Initial studies proposed that SNAP-25 aligned via an exosite orientation that was then refined to a pocket model to describe the interactions between LC/A and SNAP-25. Detailed reciprocal mutagenesis of LC and SNAP-25 defined a series of dis-contiguous interactions between SNAP-25 and LC/A. Optimal scissile bond cleavage utilized five discreet pockets of amino acids within LC/A that contribute to either efficient binding or cleavage of SNAP-25 between Q^197^ and R^198^ [44,53].

The mechanism of scissile bond cleavage for SNAP-25 by LC/A has been studied in detail and proceeds as follows. Upon SNAP-25 binding the zinc ion the coordination of zinc binding changes position which allows the carbonyl oxygen of E^197^ in SNAP-25 to displace a catalytic water molecule, allowing the water molecule to form two hydrogen bonds with E^224^, while staying weakly coordinated to the zinc ion. This intermediate state allows E^224^ which acts as a proton shuttle to perform a nucleophilic attack on the carbonyl carbon of the scissile peptide bond. This intermediate is stabilized by Y^366^ which provides the hydroxide for interaction. The last step in the cleavage of the scissile bond occurs when the intermediate state collapses and E^224^ mediates the transfer of two protons onto the scissile amide, which generates a stable amino group that then leaves the active site [55]. Understanding how the mechanism of cleavage works allows the field to optimize the efficiency of LC-based therapeutics. 

## 5. BoNT Duration and Potency

Several pathways are responsible for protein turnover in eukaryotic cells, including lysosome- and ubiquitin-mediated degradation. Lysosome-mediated proteolysis often involves the initial trafficking of cell surface proteins into endocytic vesicles that mature into endosomes and eventually fuse with lysosomes, which contain numerous degradative enzymes, including proteases [56]. An alternative to the lysosomal mediated-proteolysis is the ubiquitin-proteasome pathway, the major pathway for intracellular protein degradation in eukaryotic cells. Ubiquitin is a 76-amino acid polypeptide that is attached to proteins destined for degradation via covalent binding to K^48^ by the targeted protein, through a process using E1, E2, or two different E3s (RING or HECT) [56]. There are also chain elongation factors known as E4, which are a subclass of E3 which only catalyze chain extension of ubiquitin onto the protein [57]. Binding to E1 activates ubiquitin, which is transferred to E2. The final transfer of ubiquitin to the target protein is performed by E3. E3 (HECT) takes ubiquitin from E2 and transfers ubiquitin to the protein targeted for degradation. A second pathway is performed by E3 (RING) which mediates transfer of ubiquitin from E2 to the protein targeted for degradation without directly contacting ubiquitin [56,57]. Many proteins are polyubiquitinated, and once ubiquitinated, proteins are degraded by the proteasome into amino acids [56]. Proteins are also degraded during apoptosis, where caspases are used for mediated degradation. Caspases contain a conserved Gln-Ala-Cys-X-Gly and cleave proteins at aspartic acid. During apoptosis, caspases cleave cellular substrates such as poly (ADP-ribose) polymerase and lamins [58]. 

The average protein in a eukaryote cell has a typical half-life of ~1.5 to 48 h [59,60,61]. While a plethora of long-lived proteins (LLPs) are found in the nucleus, such as histones and nuclear pore complexes, LLPs have also been identified within other intracellular locations. The current understanding for the slow turnover of LLPs is based upon slow turnover rates when found in protein complexes or cellular structures that cannot be replaced without disrupting the complex [62]. Recently, Heo et al. characterized the half-life for proteins in the synapse, using stable isotope labeling where heavy-isotope-labeled amino acids (Lys-13-C^6^) were introduced into the diet of mice for 7 weeks and, to measure turnover, followed with a normal diet, where 164 LLPs were found in synaptosomes. In these experiments, proteins present in both the cytosol and the synaptosomes had higher turnover rates when present in the cytosol. The authors proposed that proteins in the synapse formed more stable complexes than in the cytosol which were then more accessible to protein degradation machinery [63].

**Figure 4:**
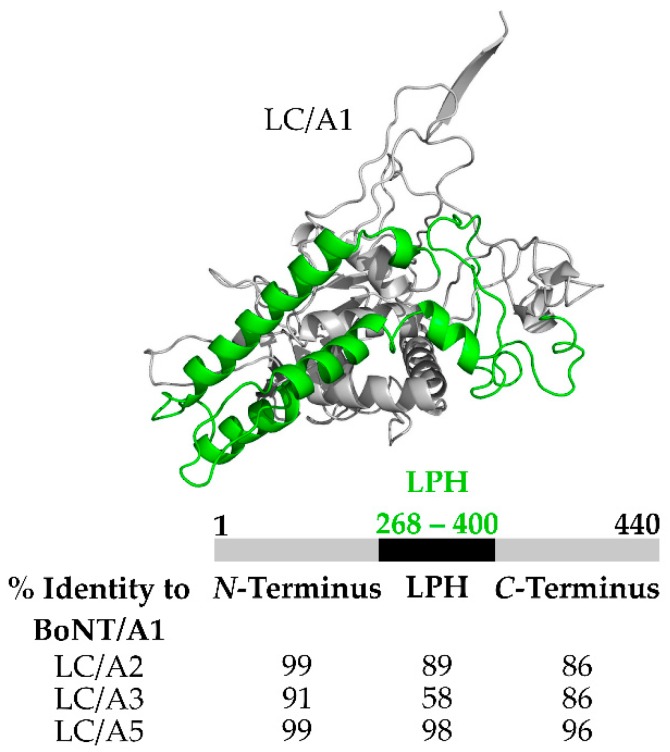
Structure and alignment of the Low Primary amino acid Homology domain (LPH) of LC/A3. (Top) Crystal structure of BoNT LC/A1 with LPH highlighted in green PDB:3BTA. (Bottom) Percent (%) homology for domains found in LC/A2, LC/A3, and LC/A5 compared to LC/A1. *N*-terminal and C-terminal regions of high homology. Low primary amino acid homology (LPH) spans residues 268–400 for LC/A1, /A2, and /A5 while residues 268–396 for LC/A3. For sequence alignment used blastp suite, with accession numbers from UniProt BoNT/A1: A2I2U2, BoNT/A2: Q84GH1, BoNT/A3: Q3LRX, BoNT/A5: C7BEA8.

BoNT duration of action is unique to each serotype and appears to be a LC function [64], ranging from weeks to several months, [65]. In general, LC duration of action in mice and human cells for BoNT/A and BoNT/C are longer than LC duration of BoNT/B, /D, /F, and /G which are longer than the duration than LC of BoNT/E (Table 1) [66]. BoNT/A has eight subtypes (A1–A8) in the current PubMed database that differ in amino acid homology up to 9.5%, have different durations of action [67,68], but each LC subtype cleaves SNAP-25 at the same location. Pellett et al. reported that primary rat spinal cord cells intoxicated with BoNT/A1, /A2, and /A5 had 50% of uncleaved SNAP-25 after 9 months, while primary rat spinal cord cells intoxicated with BoNT/A4 had 25% uncleaved SNAP-25 after 9 months [45]. In addition, BoNT/A4 had 10^3^-fold reduced activity compared to BoNT/A1 [69]. Unexpectedly, BoNT/A3 elicited a shorter half-life of duration than other BoNT subtypes where at 5 months post primary rat spinal cord cell intoxication cleaved SNAP-25 was not observed [45]. Recently, utilizing BoNT/A subtype chimeras, the lower duration of BoNT/A3 action was localized to LC action. A structure-based alignment showed that LC/A3 had a region comprising amino acids 268–396 with only ~58% amino acid homology relative to LC/A1, LC/A2, and LC/A5 (Figure 4) which was termed the Low Primary amino acid Homology region (LPH). Utilizing GFP-LC fusion proteins as a reporter, GFP-LC/A1 was found to localized to the host cell plasma membrane, while GFP-LC/A3 was present in the cytosol [64,70]. Thus, intracellular localization may contribute to the duration of BoNT/LC duration. Earlier studies showed that the *N*-terminal 17-amino acids of LC/A1 contributed to LCA1 membrane localization [71]. Thus, two or more regions may contribute to the targeting of LC/A1 to the cell membrane. 

BoNT/B includes seven subtypes that share 93% primary amino acid homology [72]. The duration of BoNT/B action ranged from 2–4 months [73,74,75,76]. BoNT/C (also known as C1) has a duration of action that approaches BoNT/A, ranging from 4 to 6 months [73]. Native chimeras of BoNT/C and BoNT/D, termed BoNT/CD and BoNT/DC may help decipher the basis for the varied range of BoNT action [77]. BoNT/D duration of action is short, ranging in weeks and BoNT/D had a relatively short duration of action in human neurons, lasting ~3 weeks post-intoxication. BoNT/D also has a lower toxicity relative to BoNT/A [78]. Each of the 14 subtypes of BoNT/E have a short duration of action in human neurons, lasting from 2–3 weeks [45,79]. Like LC/A3, LC/E is present in the cytosol when expressed in eukaryotic cells [70]. Based upon these correlates, we hypothesize intracellular localization may contribute to the duration of LC action. BoNT/F has 8 subtypes (1–8) that differ in amino acid homology by up to 30.9% [79]. In rats, BoNT/F had a shorter duration than BoNT/A [80]. The last of the novel serotypes is BoNT/G of which there are not any known subtypes and has a similar duration of action as BoNT/A and BoNT/B [81]. 

Depending on the serotype, BoNTs have different specific toxicities. Most studies determine specific toxicities in the lab, using animal models of intoxication, typically using oral or injection in mice. By intraperitoneal (injection into the gastrocnemius muscle) or intravenous data, the specific toxicity for the BoNT serotypes ranges from 1.1–1.2 ng for BoNT/A, BoNT/B, BoNT/C1, and BoNT/E to 0.4 ng for BoNT/D (Table 1) [82,83,84,85,86]. While BoNT/F, which is the least toxic of the serotypes tested, with a lethal dose of 2.4 ng (Table 1) intravenous intoxication [87]. BoNT/G toxicity is not known at this time. 

BoNT/A is the most widely studied serotype and several subtypes have been characterized for their specific toxicity. The specific toxicity of BoNT/A subtypes are similar to BoNT/A1 which is between 1 × 10^8^–2 × 10^8^ LD_50_/mg in mouse models of botulism. However, [88] BoNT/A4 has a specific toxicity of 1.0–1.25 × 10^5^ LD_50_/mg [69]. Continued analysis should determine the specific toxicity for the other subtypes of BoNT/A as well as other BoNT serotypes. Understanding the basis for LC localization may help define the duration of LC, while toxicity does not seem to be correlated to LC localization. 

## 6. Evading Host Degradation

The basis for the longevity of BoNTs are not fully understood, but several BoNT serotypes have been studied, including BoNT/A, more specifically LC/A. Tsai et al. addressed why LC persisted for long periods of time. Tsai found that a region in the C-terminal of LC/A was able to bind a deubiquitinating enzyme (VCIP135). This interaction stabilized and rescued LC from degradation by the proteasome [91]. BoNT/E has a duration of action lasting only weeks compared to BoNT/A which lasts several months. Tsai et al. studied how BoNT/E was degraded and noticed in the presence of MG132 a proteasome inhibitor, the LC of BoNT/E accumulated [92]. From this, Tsai determined that LC/E was ubiquitinated and using mass spectroscopy determined that TNF-associated factor 2 a RING E3 ligase was responsible for LC ubiquitination [92]. Shi et al. showed that when ubiquitinated, BoNT/B has decreased biological activity compared to non-ubiquitinated BoNT/B [93]. Shi also showed that in the presence of expoxomicin, a proteasome inhibitor, ubiquitinated LC/B increased compared to the phorbol 12-myristate 13 acetate (PMA) treated SH-SY5Y cells [93]; PMA is a ubiquitination enhancer [94]. Thus, ubiquitination appears to contribute to the LC longevity. Understanding how the LC avoids degradation will allow the modulation of LC duration for therapeutic application. 

## 7. BoNT Engineering 

A subfield of BoNT engineering has emerged to enhance the beneficial therapeutic potential of BoNT [64]. Pellett et al. recombined LC and HC domains of different subtypes of BoNT/A to create hybrid toxins that comprised reciprocal LC-HC components with potentially new properties but retain therapeutic and vaccine sensitivities of the parental proteins. Based upon the differential duration of toxin action, Pellett et al., characterized BoNTA1/A3 and BoNTA3/A1 chimeras [64]. While BoNTA1/A3 had greater potency than BoNTA3, showing a role for HC in potency, BoNTA3/A1 had a slight reduction in potency relative to BoNT/A1, but retained the short duration of action of BoNT/A3. Thus, this experiment showed the dominance of the HC as rate-limiting for the specific activity of botulism and that LC contributed to the duration of action in this mouse model [64]. These experiments show the utility of BoNT chimeras to define the molecular and cellular aspects of BoNT action, as tools for next generation BoNT therapies. 

BoNT engineering has also changed the substrate specificity based upon understanding LC substrate binding. LC/E substrate specificity was extended to cleave SNAP-23, by changing residue 224 of LC/E D→K (D^224^K). SNAP-23 is a nonneuronal isoform of SNAP-25, which is involved with several cellular processes, such as membrane repair, cytokinesis, and synaptic transmission [95,96,97]. The kinetics of SNAP-23 cleavage by LC/E (D^224^K) were similar to LC/B cleavage of VAMP-2, ~17 S^−1^ which is 5-fold lower than wild-type LC/E cleavage of SNAP-25. LC/E (D^224^K) cleavage of SNAP-23 in Hela cells inhibited TNF-α-mediated mucin and IL-8 secretion. Inhibiting mucin and IL-8 by LC/E (D^224^K) shows potential therapy for several human diseases that involve hypersecretion such as asthma and inflammatory disease [98], therapeutics outside the scope of neurological conditions. Recently, Binz et al. mapped the region that is responsible for making human SNAP-23 resistant to cleavage by LC/A at physiological concentrations. Mutating 10 amino acids in SNAP-23 (between residues 172–206) yielded a substrate that was cleaved by LC/A with similar properties as SNAP-25. Re-engineering LC/A was also performed, using a yeast-based screening system to identify residues involved in the P2′ pocket recognition. The LC/A(A^308^V) mutation was 2.5-fold more efficient for SNAP-23 cleavage than wild-type LC/A. Although more experiments are needed to further understand the rate limiting steps, both in catalysis and binding, for LC/A cleavage of SNAP-23, these mutations are a significant advancement towards the development of pharmaceuticals for use to treat non-neuronal based diseases of hypersecretion [99]. 

Due to BoNTs neuronal specificity for entry into the presynaptic membrane of neurons within synaptic vesicles as well as the trafficking of LC to the inner leaflet of the plasma membrane or synaptic vesicles, an emerging field of study is the use of BoNT as a vehicle for therapeutic delivery inside a cell. Dolly and coworkers have engineered various BoNT-derivatives with substitute host and bacterial factors that have provided insight into BoNT action and the use of BoNT as conjugate carriers [100,101]. Vazquez-Citron et al. designed an atoxic derivative of BoNT/C1, which possessed the same key entry characteristics of wild-type BoNT/C1, while being 5 × 10^6^-fold less toxic than wild-type BoNT/C1 [102]. While the use of BoNT as a vehicle is promising, a caveat to this technique is the assurance that the vehicle has a large enough therapeutic range that toxicity will not be a factor. Naturally occurring BoNTs are encased by progenitor toxin complexes, which are high molecular weight multi-protein complexes composed of BoNT and several non-toxic neurotoxin-associated proteins (NAPs) [103]. One NAP is a non-toxic non-hemagglutinin (NTNHA) protein [104,105]. To study this progenitor toxin complex, BoNT/A1 was engineered with a three-point mutation in the LC (E^224^Q/R^363^A/Y^366^F). Gu et al. added a BoNT/A-specific nanobody which improved crystal packing and diffraction quality. Progenitor toxin complex crystal structure provided orientation and identified interactions between the two complexes. A better understanding of the progenitor toxin complex could render the complex as a drug delivery system [106]. 

Use of the current chemically inactivated BoNT vaccine has been discontinued based upon the loss of vaccine potency [107]. Recently, Webb et al. engineered full-length BoNT/A (H^223^A, E^224^A, H^227^A) as a vaccine candidate that was more potent than the HCC based vaccine. Note, the H^223^A, E^224^A, H^227^A mutations lie within the Zn binding motif of the LC (Figure 1) [108]. A second vaccine strategy, to further reduce residual toxicity and potential reversion, engineered recombinant, full length BoNT/A with three-point mutations within the LC (E^224^A, R^363^A, Y^366^F), along with a point mutation in the ganglioside binding pocket W^1226^A [109], which also proved to be a potent vaccine candidate. Recombinant, full length BoNT vaccines, inactivated via mutations in LC function, may provide a platform towards the development of an FDA-approved vaccine. 

## 8. Conclusions

This review addresses the molecular, cellular, and structural properties of LCs with respect to toxicity and duration of BoNT action, which provide the basis for BoNT engineering and BoNT vaccine production. Advances in understanding LC action also provides the basis for BoNT as a therapeutic and as a delivery platform. 

## Figures and Tables

**Table 1:** Toxicity and duration of all seven BoNT serotypes (A–G).

Serotype ^1^	Toxicity	Duration
/A	1.2 ng [82]	<9 months [45]
/B	1.2 ng [83]	2–4 months [73,74,75,76]
/C1	1.1 ng [89]	4–6 months [73]
/D	0.4 ng [85]	~3 weeks [78]
/E	1.1 ng [86]	2–3 weeks [45,79]
/F	2.5 ng [87]	<A1 [80]
/G	120 LD_50_/kg ^2^ [90]	n.d.

^1^ intravenously administered or intraperitoneal administered of the indicated BoNT. Toxicity = the amount of toxin required to kill 50% of intoxicated mice (LD_50_). Duration = time following exposure to an equivalent dose of the indicated BoNT when 50% of substrate is uncleaved. n.d. is not determined (not known at the time of review). ^2^ The lethal dose for mouse LD_50_ per kg intravenously administered.
